# Antibiotic binding releases autoinhibition of the TipA multidrug-resistance transcriptional regulator

**DOI:** 10.1074/jbc.RA120.016295

**Published:** 2020-10-22

**Authors:** Xuguang Jiang, Linjuan Zhang, Maikun Teng, Xu Li

**Affiliations:** 1 Hefei National Laboratory for Physical Sciences at Microscale, School of Life Sciences, National Synchrotron Radiation Laboratory, University of Science and Technology of China, Hefei, Anhui, China; 2 Department of Cell Biology and Anatomy, Graduate School of Medicine, University of Tokyo, Tokyo, Japan

**Keywords:** multidrug resistance (MDR), transcriptional regulator, TipA, autoinhibition, drug resistance, antibiotic resistance, transcription regulation, transcription promoter, DNA-binding protein, crystal structure, activation mechanism, structure biology

## Abstract

Investigations of bacterial resistance strategies can aid in the development of new antimicrobial drugs as a countermeasure to the increasing worldwide prevalence of bacterial antibiotic resistance. One such strategy involves the TipA class of transcription factors, which constitute minimal autoregulated multidrug resistance (MDR) systems against diverse antibiotics. However, we have insufficient information regarding how antibiotic binding induces transcriptional activation to design molecules that could interfere with this process. To learn more, we determined the crystal structure of SkgA from *Caulobacter crescentus* as a representative TipA protein. We identified an unexpected spatial orientation and location of the antibiotic-binding TipAS effector domain in the apo state. We observed that the α6–α7 region of the TipAS domain, which is canonically responsible for forming the lid of antibiotic-binding cleft to tightly enclose the bound antibiotic, is involved in the dimeric interface and stabilized via interaction with the DNA-binding domain in the apo state. Further structural and biochemical analyses demonstrated that the unliganded TipAS domain sterically hinders promoter DNA binding but undergoes a remarkable conformational shift upon antibiotic binding to release this autoinhibition via a switch of its α6–α7 region. Hence, the promoters for MDR genes including *tipA* and RNA polymerases become available for transcription, enabling efficient antibiotic resistance. These insights into the molecular mechanism of activation of TipA proteins advance our understanding of TipA proteins, as well as bacterial MDR systems, and may provide important clues to block bacterial resistance.

From bacteria to humans, cells employ multidrug resistance (MDR) systems to respond to and scavenge a wide variety of structurally and chemically diverse cytotoxic compounds (1, 2). In addition to the well-known efflux pump-mediated MDR mechanisms, cytoplasmic-soluble proteins are also able to recognize and sequester many toxic compounds or drugs (3, 4). The expression of both MDR transporters and cytoplasmic responders is often regulated by drug-sensing transcription factors that directly recognize and interact with the corresponding substrates (5, 6). By the combined chemoprotective actions of MDR proteins and soluble drug sensors, cytotoxic compounds are promptly detected and effectively removed from the interiors of cells (5, 7). Multidrug (MD) recognition by MDR and regulatory sensor proteins is often achieved in an adaptable and versatile MD-binding pocket with multiple binding capabilities for diverse ligands, and global conformational transformations induced by ligand-pocket binding promote active transport or transcription activation (8–11).

The TipA (thiostrepton-induced protein A) class of transcriptional regulators found in diverse bacteria is viewed as a minimal autoregulated MDR system (12–14). TipA proteins belong to a subset of the superfamily of mercuric ion resistance (MerR)–like transcriptional regulators (15, 16) that contain an N-terminal helix-turn-helix (HTH) motif followed by an intermediate linking coiled-coil (PF00376 MerR and PF13411 MerR_1 families in the Pfam database) and a C-terminal TipAS-type effector domain (PF07739 TipAS family in the Pfam database) (Fig. 1*A*). Investigation of *Streptomyces lividans* had revealed that expression of the *tipA* gene with two alternative start codons produces two protein isoforms: a full-length transcriptional regulatory TipAL protein and a short TipAS protein consisting only of the C-terminal domain of TipAL (17–19). The TipAS protein is the predominant form that is capable of recognizing thiopeptide compounds via covalent binding by an active cysteine (13, 20), which then induces structural reordering to form a large hydrophobic cleft for permanent sequestration and neutralization of the antibiotics (14, 18). However, upon thiopeptide binding into the C-terminal TipAS domain, the N terminus of TipAL (TipAN) is activated to bind promotors via the HTH domain and promote transcriptional activation of multiple genes, including *tipA* for antibiotic resistance (14, 21).

**Figure 1.**
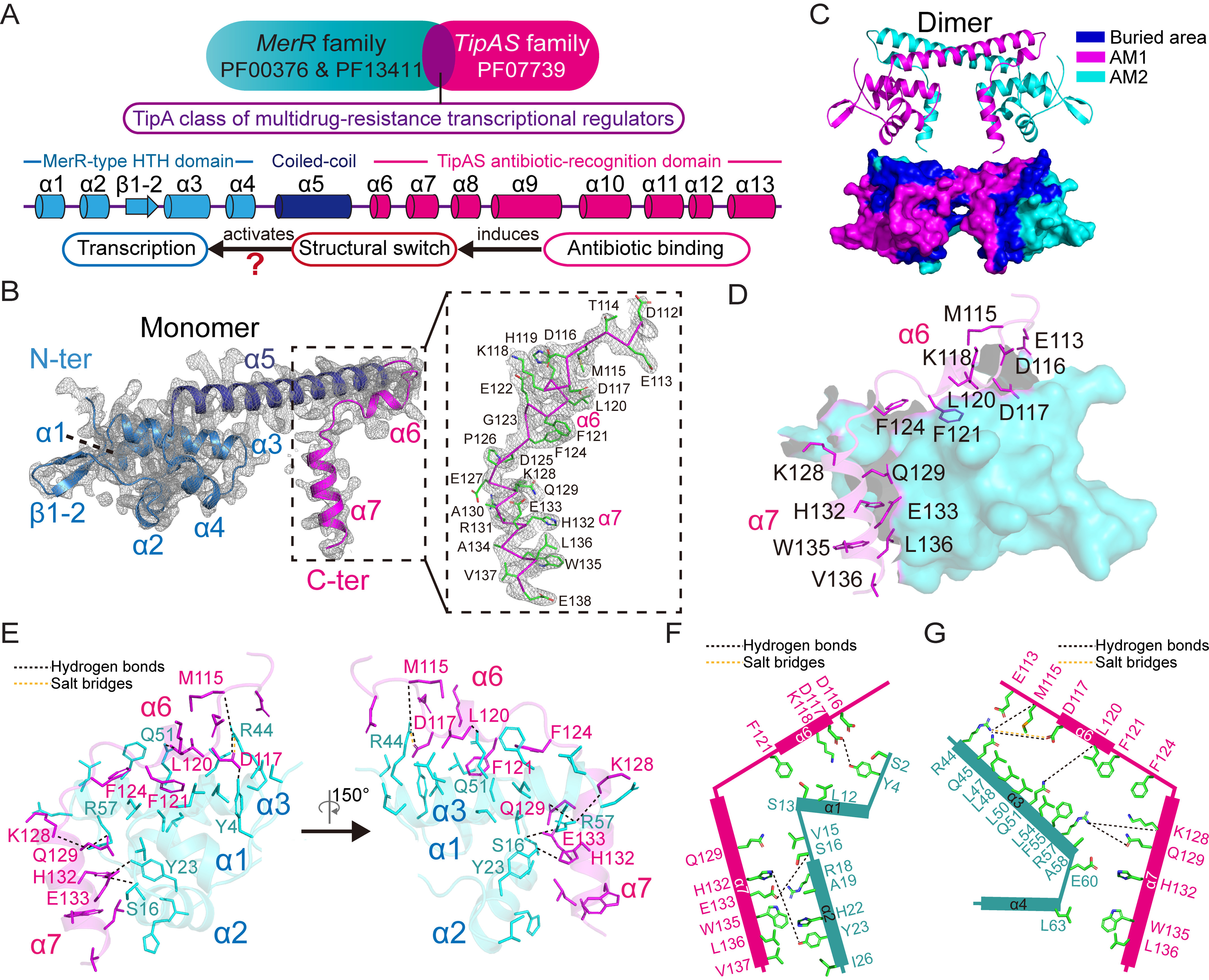
**Crystal structure of *C. crescentus* TipA-class protein SkgA.** *A*, schematic diagrams showing the domain composition of the TipA-class regulators. The structure of TipA proteins is constituted mostly by α**-**helices with initial four helices within the DNA-binding domain, an intermediate coiled-coil α5 helix responsible for the dimerization, and the rest of the eight helices composing the TipAS domain. *B*, crystal structure of SkgA with one molecule in the asymmetric unit. The 2*F*_o_ − *F*_c_ density map is contoured at 1σ. The α6–α7 region is highlighted in the *right panel* with ribbon representation for main chain and stick representation for side chain. *N-ter*, N terminus; *C-ter*, C terminus. *C*, dimeric structure of SkgA that is constructed by molecules from two adjacent asymmetric units. The protein surface is shown in the *lower panel* with buried area colored in *blue*. *AM*, asymmetric molecule. *D*, the α6–α7 region is involved in the dimeric interface. The α6–α7 region of one protomer interacted with the N-terminal DNA-binding domain of the other protomer. Amino acid residues of the α6–α7 region involved in the interaction are labeled with side chains shown as *sticks*. *E*, interactions between the α6–α7 region and N-terminal DNA-binding domain. Hydrogen bonds and salt bridges formed between residues are indicated by *black* and *yellow dotted lines*, respectively. The other labeled residues are involved in hydrophobic interactions. *F*, schematic diagram showing the interactions between the α6–α7 region and α1–α2 region in the SkgA dimer. *G*, schematic diagram showing the interactions between the α6–α7 region and α3–α4 region in the SkgA dimer.

In addition to *S. lividans*, TipA-class proteins have also been reported to have crucial regulatory functions in other species such as Mta (multidrug transporter activation protein) from *Bacillus subtilis* (22), stationary-phase regulation of KatG protein (SkgA) from *Caulobacter crescentus* (23), and AlbA (albicidin resistance protein) containing two tandem TipAS domains from *Klebsiella oxytoca* (21, 24). Structural analyses using apo and *mta* promoter-bound structures of the Mta N terminus (MtaN) revealed that MtaN possesses a canonical MerR-type HTH motif that binds promoter DNA to activate transcription using a distortion mechanism identical to BmrR, which belongs to another MerR-type MDR gene regulator family with a GyrI-like effector domain (25, 26). However, because of the lack of ligand-bound full-length structural information, whether TipA-class regulators also adopt a drug-induced activation mechanism similar to BmrR, which was elaborated by the analysis of the liganded BmrR–*bmr* promoter complex structure, remains unknown (27–29).

Previous NMR studies with accurate determinations of apo and liganded TipAS proteins indicated that thiopeptide binding induces stabilization and restructuring of the flexible N-terminal region and thus results in the formation of a large MD-binding pocket (14, 18). Nevertheless, it is still a missing part in the current model of how this kind of drug-induced change in the TipAS domain drives a global structural shift to facilitate transcriptional activation of TipAN in the context of full-length TipAL protein (Fig. 1*A*).

Here, we sought to obtain full-length structural information of TipA-class proteins, and the SkgA structure was successfully determined at a resolution of 2.5 Å. The solved structure indicated a significantly different orientation of the TipAS effector domain compared with the GyrI-like effector domain of BmrR. Structural and biochemical analyses indicated that the interaction of TipA regulators with promoter DNA is hindered in the apo state and revealed an unexpected activation mechanism in which drug binding induces conformational turnover of the TipAS domain to release the N-terminal HTH motif for promoter DNA binding and subsequent transcriptional activation, during which the α6–α7 region may serve as a key switch element to drive the global transition. Remarkably, further biochemical results strongly support the above conclusion by showing that mutations on the drug-recognizing sites within the α6–α7 region resulted in no significant loss of drug-binding affinity but a sharp reduction of promoter DNA-binding affinity. Overall, our findings provide a crucial complement to the current understanding of TipA-class MDR systems.

## Results

### Basic architecture and dimeric interaction of C. crescentus TipA-class protein SkgA

Full-length proteins of three reported TipA-class transcriptional regulators, *S. lividans* TipAL, *C. crescentus* SkgA, and *B. subtilis* Mta were expressed, purified, and set up for crystallization. As a result, diffraction-quality crystals of SkgA were obtained, and the structure was determined at a resolution of 2.5 Å using the single-wavelength anomalous dispersion phasing method (Table 1). SDS-PAGE analysis indicated that the crystals were composed of full-length SkgA proteins (Fig. S1*A*). Although full-length SkgA (residues 1–255) protein was used for crystallization, only residues 1–138 were observed in the final structure. The established structure consists of the N-terminal MerR-type HTH motif, coiled-coil region, and the initial two α-helices of the C-terminal TipAS domain (Fig. 1*B* and Fig. S1*B*), whereas the rest of the TipAS domain was still invisible in the current model, probably because of structural flexibility or diffraction damage. As expected, the amino acid sequences of the N-terminal MerR-like DNA-binding domain and coiled-coil region of SkgA share a high level of identity with those of other TipA-class proteins and BmrR (Fig. S1, *C* and *D*).

**Table 1:** **Data collection and refinement statistics**

	SkgA	SeMet-SkgA
**Data collection statistics**		
X-ray source	Shanghai Synchrotron Radiation Facility Beamline 17U1	Shanghai Synchrotron Radiation Facility Beamline 17U1
Space group	P6522	P6522
Unit cell parameters		
*a*, *b*, *c* (Å)	89.57, 89.57, 88.96	89.61, 89.61, 89.22
α, β, γ (°)	90.00, 90.00, 120.00	90.00, 90.00, 120.00
Wavelength (Å)	0.9785	0.9785
Resolution limits (Å)*^a^*	50.00–2.50	50.00–2.90
No. of unique reflections	7759	4765
Completeness (%)	95.8 (80.5)	94.9 (99.2)
Redundancy	32.8 (13.1)	34.0 (29.4)
*R*_merge_ (%)*^b^*	11.1 (114.5)	21.0 (193.6)
*R*_p.i.m._ (%)	1.9 (29.9)	3.7 (35.9)
Mean I/σ (I)	37.8 (1.75)	23.0 (2.0)
**Refinement statistics**		
Resolution limits (Å)	50.0–2.50	
*R*_work_ (%)*^c^*/*R*_free_ (%)*^d^*	21.7/26.9	
RMSD		
Bonds (Å)	0.015	
Angles (˚)	1.765	
Mean B factor (Å^2^)	40.8	
No. of nonhydrogen protein atoms	1117	
No. of water oxygen atoms	52	
Ramachandran plot (%)		
Most favored regions	97.08	
Additional allowed regions	2.92	
**PDB entry**	7CLA	

*^a^* The values in parentheses are for the highest resolution shell.

*^b^ R*_merge_ = Σ*_h_* Σ*_l_* |*I_hl_* − <*I_h_*>|/Σ*_h_* Σ*_l_* <*I_h_*>, where *I_hl_* is the *l*th observation of reflection *h*, and <*I_h_*> is the weighted average intensity for all observations l of reflection *h*.

*^c^ R*_work_ factor = Σ*_h_*‖*F*_obs(_*_h_*_)_ | − |*F*_cal(_*_h_*_)_‖/Σ*_h_*|*F*_obs(_*_h_*_)_|, where *F*_obs(_*_h_*_)_ and *F*_cal(_*_h_*_)_ are the observed and calculated structure factors for reflection *h*, respectively.

*^d^ R*_free_ factor was calculated same as *R*_work_ factor using the 5% the reflections selected randomly and omitted from refinement.

Because MerR family proteins reportedly function as dimers and SkgA protein also exhibits a dimer conformation in solution (Fig. S2*A*), a dimeric SkgA structure was established by combining two molecules from two adjacent asymmetric units for subsequent analysis (Fig. 1*C* and Fig. S2*B*). The α6–α7 region of the SkgA dimer does not show any significant crystal contact with other symmetry mates (Fig. S2*C*). The dimeric interface was analyzed by the programs PyMOL and PISA (30). The total surface area of the SkgA dimer is 15,051.4 Å^2^, and the buried area is 5,404.7 Å^2^ (Fig. 1*C* and Fig. S2*B*). Remarkably, apart from the coiled-coil α5 helices, the α6–α7 region and N-terminal domain are also involved in the dimeric interface. Fifteen residues of the α6–α7 region of each protomer interact with the N-terminal domain of the other protomer via hydrogen bonding, ionic bonding, and hydrophobic interactions (Fig. 1, *D–G*), which contribute to stabilization of the conformation of the α6–α7 region. Eleven residues of the N-terminal α1–α2 region exhibit interactions with ten residues of the α6–α7 region via hydrophobic interactions and three hydrogen bonds formed by residue pairs Tyr^4^–Asp^117^, Ser^16^–Glu^133^, and Tyr^23^–His^132^ (Fig. 1, *E* and *F*). Twelve residues from the N-terminal α3–α4 region interact with eleven residues of the α6–α7 region via hydrophobic interactions, a salt bridge of Arg^44^–Asp^117^, and four hydrogen bonds of Arg^44^–Met^115^, Gln^51^–Leu^120^, Arg^57^–Lys^128^, and Arg^57^–Gln^129^ (Fig. 1, *E* and *G*).

**Figure 2.**
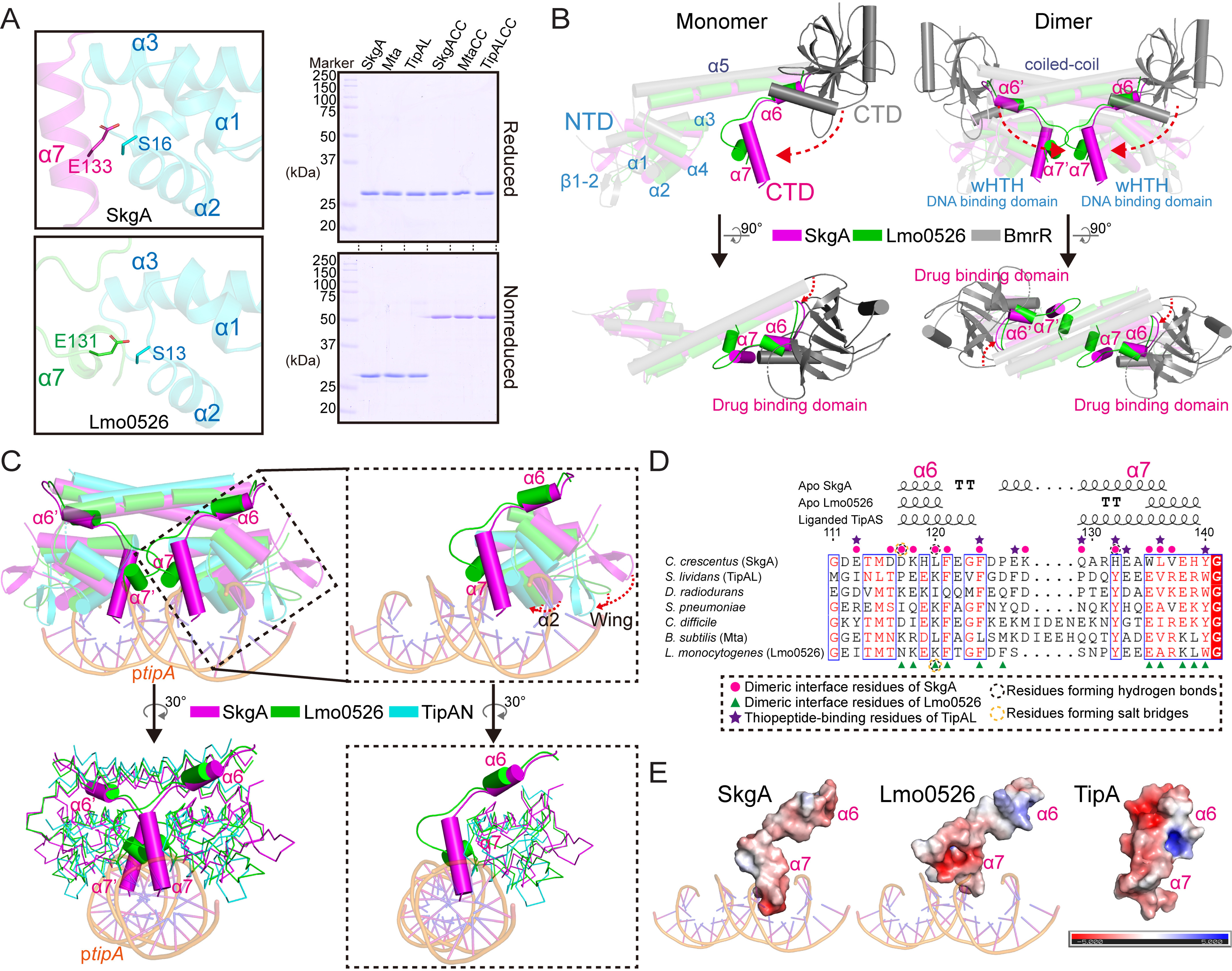
**Structural analysis revealed a potential inhibition of the TipA–DNA interaction by the conserved α6–α7 region.** *A*, in-solution validation of the specific conformation of the α6–α7 region shown in crystal structure. Ser^16^ and Glu^117^ that are involved in the dimeric interface exhibit close location in the structure of the SkgA dimer; so are their corresponding residues Ser^13^ and Glu^131^ in the Lmo0526 structure (*left panel*). Formation of disulfide bonds of mutant and WT SkgA, Mta, and TipAL was analyzed using reduced and nonreduced SDS-PAGE (*right panel*). *B*, structural superposition of TipA-class proteins with BmrR indicated a distinctive spatial location of TipAS domain in the apo state. Cα atoms of each structure were used for the superposition in the PyMOL. The significant structural discrepancy on the orientation of C-terminal effector domains between TipA-class proteins and BmrR is indicated by *red dashed arrows*. *NTD*, N-terminal domain; *CTD*, C-terminal domain; *wHTH*, winged helix-turn helix motif. *C*, structural superposition of apo SkgA and Lmo0526 with promoter DNA-bound TipAN (PDB code 2VZ4). Structures are represented as cartoons with cylindrical helices in the *upper panel* and as ribbons except for the α6–α7 region in the *bottom panel*. Close-up views highlighting the α6–α7 helices and DNA are shown in the *right panel*. The putative shifts of α2 helix and wing region upon DNA binding are indicated by *red dashed arrows*. *D*, structure-based sequence alignment of the α6–α7 regions from TipA-class regulators. Amino acid numbering is given according to *C. crescentus* SkgA, and the corresponding second structural elements of SkgA, Lmo0526, and TipA were given above the sequences. The second structure of TipA was shown according to the liganded TipAS structure (PDB code 2MC0) with stabilized α6–α7 helices. Residues involved in the dimeric interface of SkgA and Lmo0526 are indicated by *magenta circles* and *green arrowheads*, respectively. Thiopeptide-binding residues of TipA are indicated by *purple stars*. Residues forming hydrogen bonds and salt bridges are indicated by *black* and *yellow circled dots*, respectively. *E*, electrostatic distribution of α6–α7 regions of TipA, SkgA, and Lmo0526. The liganded TipAS structure (PDB code 2MC0) was used here for the analysis of TipA. The APBS plugin within the program PyMOL was used for calculations of charge distribution.

Similar results were found in the structure of *Listeria monocytogenes* TipA-class protein Lmo0526 with modeled α6–α7 helices (PDB code 3QAO) (Fig. S3*A*). The α6–α7 region of Lmo0526 also exhibits a wide interaction with the N-terminal DNA-binding domain in the Lmo0526 dimer via hydrogen bonding, ionic bonding, and hydrophobic interactions (Fig. S3, *B*–*E*), which is in line with the corresponding interaction in the SkgA dimer. The α6–α7 region of the Lmo0526 dimer also does not exhibit any significant crystal contact with other symmetrical molecules (Fig. S3*F*). Notably, many of these interacting residues are conserved among the TipA-class family proteins, and the corresponding residues of some antibiotic-binding residues reported in TipA and DNA-binding residues revealed by MtaN–DNA and TipAN–DNA structures are involved in the interaction (Fig. S4). Collectively, these findings suggested that the α6–α7 region of the TipA-class proteins, which is reportedly unstructured flexible loops in unliganded TipAS protein (14, 18), is stabilized with an ordered conformation via interaction with the N-terminal domain in the apo state.

**Figure 3.**
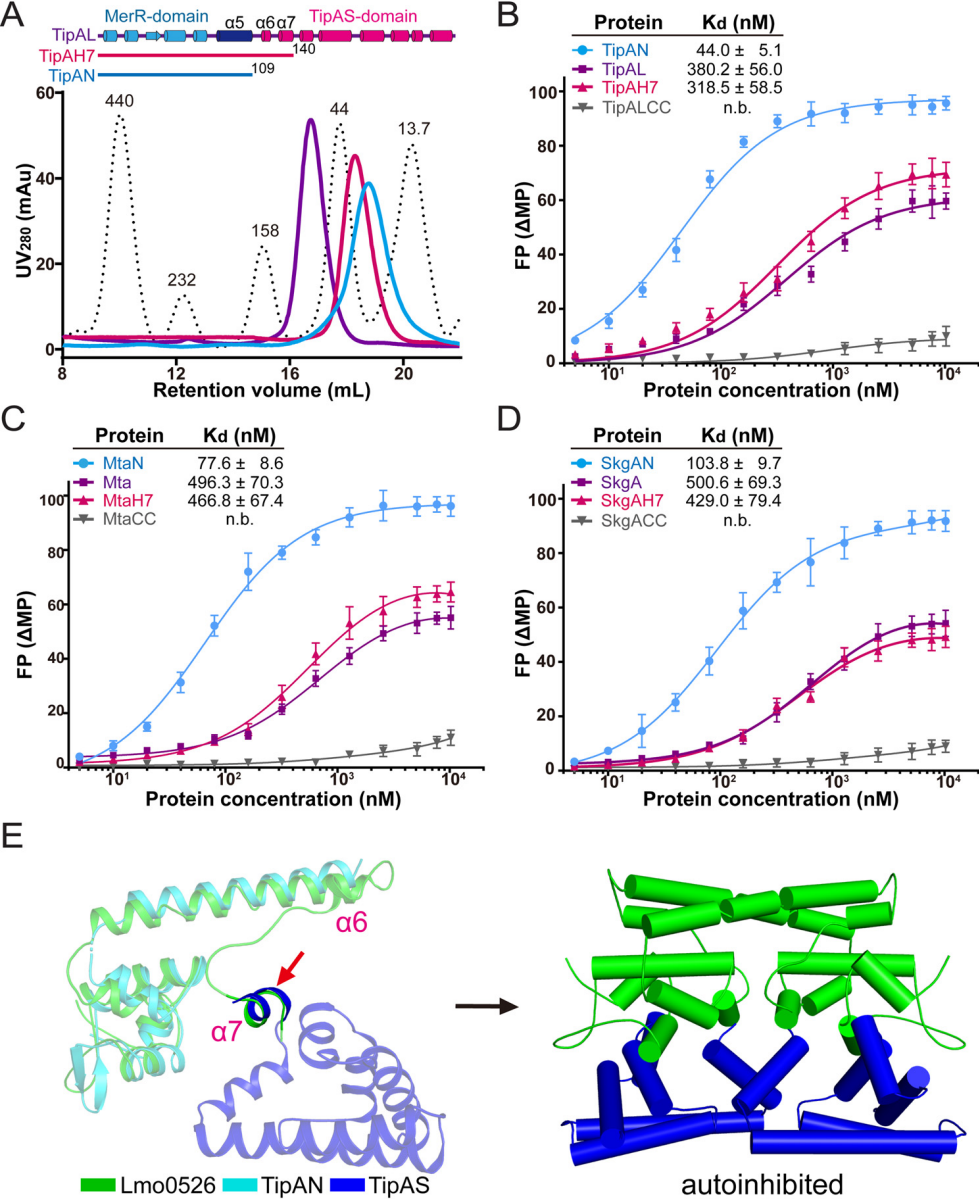
**Biochemical assessments indicated that the promoter-DNA interaction of unliganded TipA regulators is hindered by the TipAS domain.** *A*, size**-**exclusion assays analyzing full-length and truncated TipAL proteins. Full-length TipAL and two truncations, TipAN (residues 1–109) and TipAH7 (residues 1–140), all exhibited as dimers in solution. Standard marker is indicated by *dotted lines* with the molecular size (kDa) of each protein peak marked. *B–D*, FPA examining binding affinities of TipA proteins (*B*), Mta proteins (*C*), and SkgA proteins (*D*) with their respective promoter DNA. Their double-cysteine mutants were also included in the assessment. The data were plotted using a base 10 logarithmic scale for the *x* axis and were fitted with a one-site binding model using the program GraphPad Prism7. *E*, the potential location of apo-state TipAS domain revealed by structural alignment. Structures of Lmo0526 and TipAS (PDB code 2MC0) were aligned at conserved and ordered α7 helices of relatively high sequence homology (indicated by a *red arrow*), and TipAN structure was further aligned to Lmo0526 (*left panel*). By the alignment, a possible ligand-free model for full-length TipA-class proteins was established with the TipAS domain lying beneath the HTH DNA-binding domain, which represents an inactive autoinhibited conformation (*right panel*).

**Figure 4.**
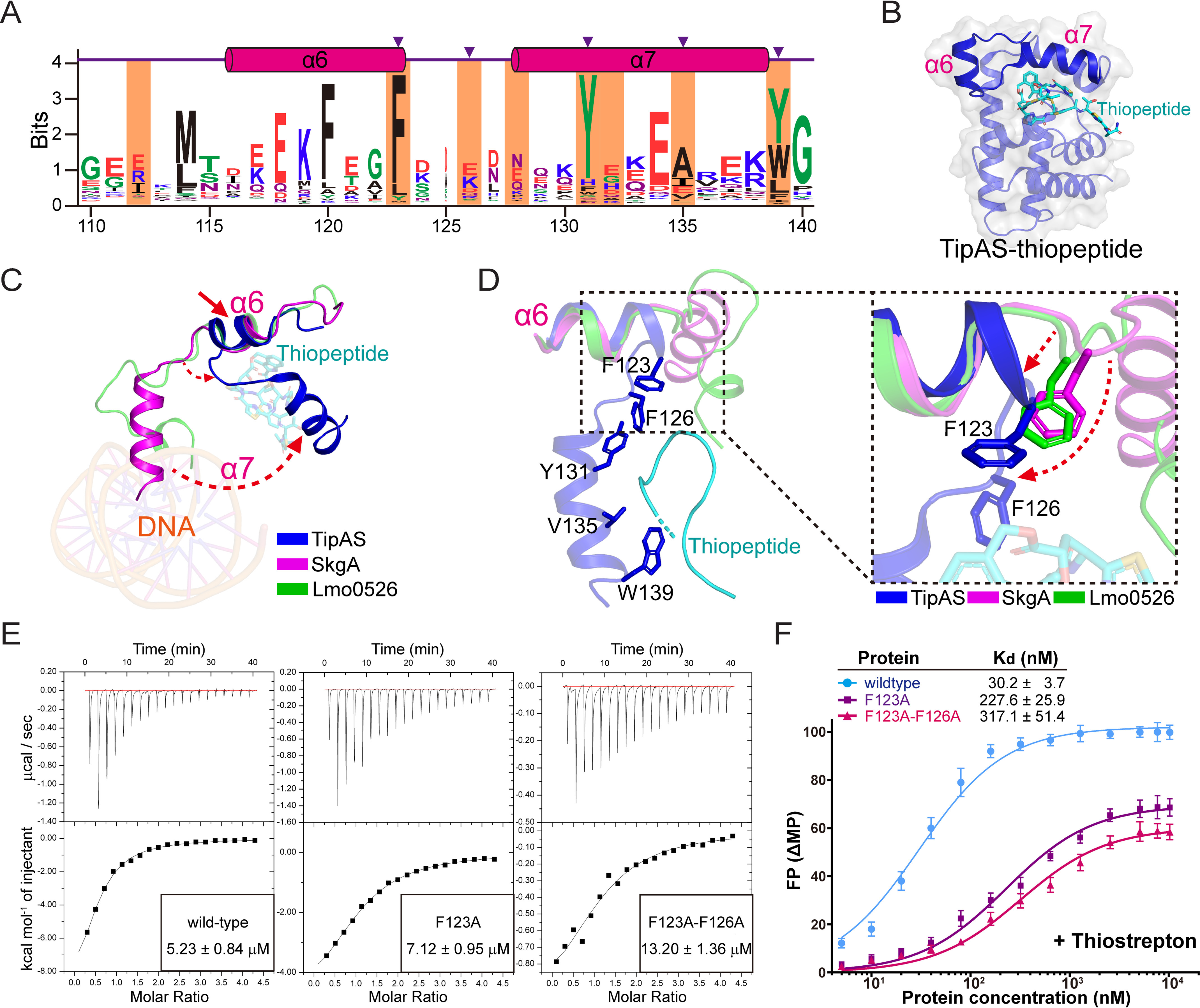
**An antibiotic-induced structural shift of α7 helix is required for transcriptional activation.** *A*, results of multiple sequence alignment of the α6–α7 region. After removal of repeated entries from the TipAS family (PF07739 in Pfam database), the remaining complete sequences were realigned by using the program Clustal Omega as an input for the alignment via WebLogo3. Amino acid numbering and helical position are given according to *S. lividans* TipA. Residues involved in antibiotic interaction are highlighted in *orange*, and key residues responsible for antibiotic recognition are indicated by *purple arrowheads*. *B*, structural representation of thiopeptide-bound TipAS (PDB code 2MC0) with the α6 and α7 helices highlighted. *C*, structural comparison of the α7 helices from SkgA, Lmo0526, and thiopeptide-bound TipAS with an alignment at the α6 helices. Potential deflections of helix and loop are indicated by *red dashed arrows*. *D*, close-up view of thiopeptide-associating residues based on the alignment at α6 helices. Phe^123^ and Phe^126^ of the liganded TipAS in the loop region are highlighted. The potential structural deflections of loop regions upon antibiotic binding are indicated by *red dashed arrows*. *E*, ITC experiments assessing the thiopeptide-binding affinities of WT and mutant (F123A and F123A/F126A) TipAL proteins. The data were processed using the program Microcal-Origin. *F*, FPA experiments examining the promoter DNA-binding affinities of the WT and mutant TipAL proteins in the presence of thiostrepton. The data were plotted using a base 10 logarithmic scale for the *x* axis and were fitted with a one-site specific binding model using the program GraphPad Prism7.

### The effector domain exhibits a distinctive orientation in TipA proteins

To confirm whether the in-solution conformation of the α6–α7 region is consistent with the conformation in crystal structures, double-cysteine mutants of TipA-class proteins were constructed and analyzed. In light of the structures, the conserved interacting residue pairs Ser^16^–Glu^133^ of SkgA and Ser^13^–Glu^131^ of Lmo0526 exhibit close distances (Fig. 2*A* and Fig. S4). Therefore, double-cysteine mutants, SkgACC (S16C/E133C), MtaCC (S15C/E138C), and TipALCC (T15C/E134C), were constructed based on sequence homology (Fig. S4) and were analyzed by both reduced and nonreduced SDS-PAGE. As a result, although WT SkgA, Mta, and TipAL were shown as monomer bands, the double-cysteine mutants were all shown as dimer bands in the nonreduced SDS-PAGE gel as a result of the formation of disulfide bonds (Fig. 2*A*, *right panel*). The results indicated that in solution, the α6–α7 region of the TipA-class protein exhibits a conformation consistent with that revealed by SkgA and Lmo0526 structures.

Alignment of the two structures indicated that the α6 helices share a high structural similarity, whereas the α7 helices differ slightly in length and direction. However, notably, in the two structures, the α6–α7 helices can be seen to consistently extend from the α5 helix toward the DNA-binding interface, indicating that the relative orientation of the TipAS domain in TipA proteins is significantly different from the relative orientation of the GyrI-like effector domain in BmrR (Fig. 2*B*).

### The α6–α7 region and TipAS domain hinder promoter DNA binding in the apo state

Because multiple DNA-binding residues are involved in the dimeric contacts with the α6–α7 helices (Fig. S4) that exhibit an unexpected orientation, we further sought to investigate how the α6–α7 region and TipAS domain with the orientation affect DNA binding. SkgA and Lmo0526 structures were aligned on Cα atoms with the B-DNA–bound TipAN structure (PDB code 2VZ4) and distorted DNA-bound MtaN structure (PDB code 1R8D), respectively. Notably, it is currently unclear whether SkgA binding to DNA will result in DNA distortion, as observed in published MerR–DNA complexes, or remain B-DNA in form, as seen in the TipAN–DNA complex. Putative shifts of the α2 and wing region required for DNA binding were indicated by the structural superposition (Fig. 2*C* and Fig. S5) as previously elaborated by structural studies on MtaN (25, 26). Importantly, the α7 helix, which is situated closely to the α2 helix, was found to occupy a part of the space for the putative bound DNA, which suggested that it may hinder both DNA loading and the movement of the α2 and wing regions for DNA binding and thus inhibit DNA association (Fig. 2*C* and Fig. S5). Analysis of the electrostatic distribution of the α6–α7 region was also performed using the two structures, as well as the NMR structure of TipAS (PDB code 2MC0). As a result, with a relatively high sequence homology (Fig. 2*D*), the α6–α7 surfaces of all three structures are strikingly electronegative, (Fig. 2*E*), which would likely impair DNA binding.

**Figure 5.**
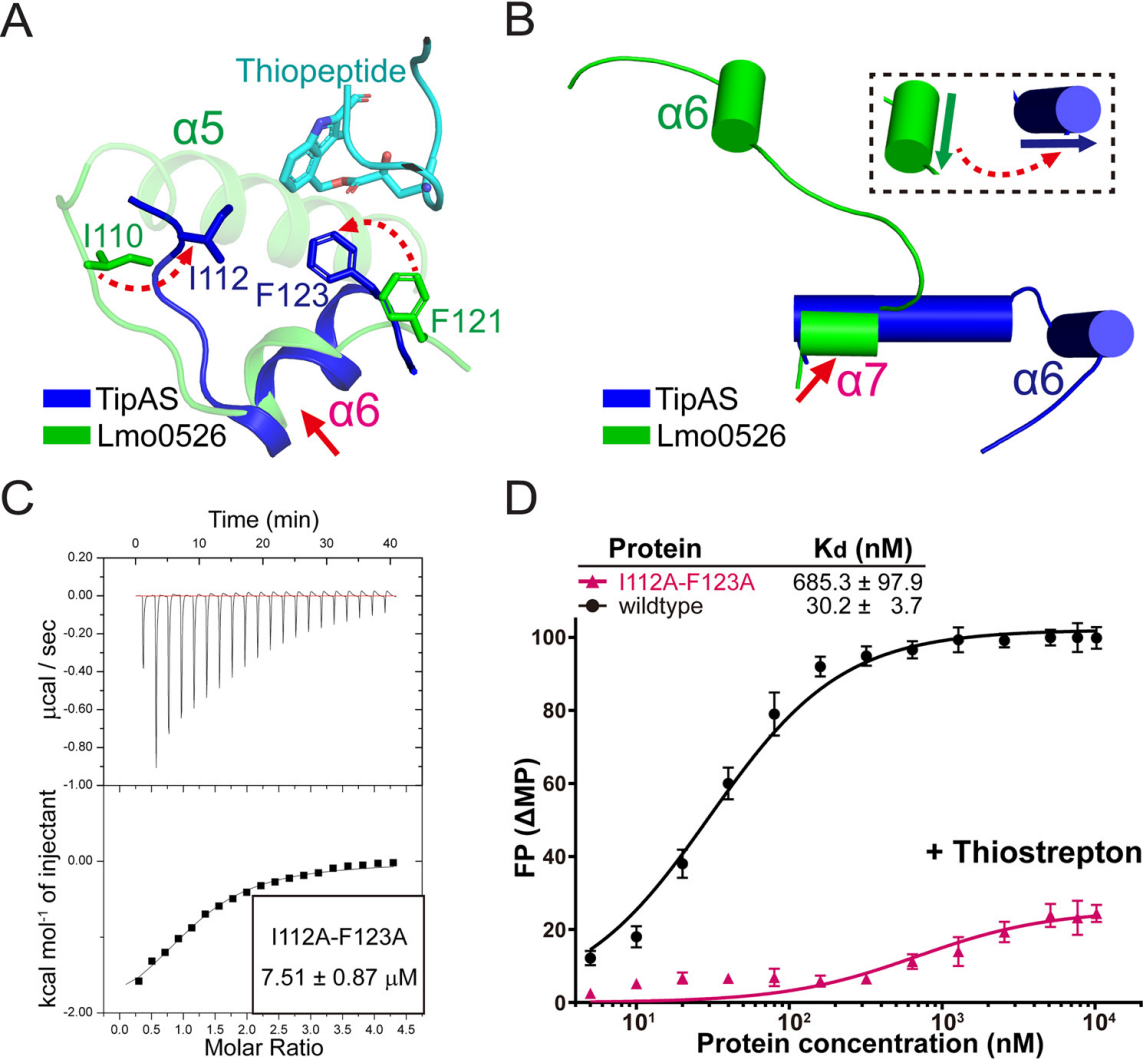
**The α6 helix may undergo a significant structural switch upon drug binding.** *A* and *B*, structural comparisons of the thiopeptide-TipAS and apo Lmo0526 structures via alignments at the α6 helices (*A*) and at the conserved structured part of the α7 helices (*B*) for probing structural discrepancies of loop regions around the α6 helix and orientational difference of the α6 helices, respectively. Two thiopeptide-interacting residues of TipAS, Ile^112^ and Phe^123^, were highlighted for a comparison with their corresponding residues in Lmo0526. The aligned regions are indicated by *red arrows*, and the significant dissimilarities are indicated by *red dashed arrows*. *C*, ITC analysis of thiopeptide-binding affinity of I112A/F123A mutant of TipAL. The data were processed using the program Microcal-Origin. *D*, FPA analysis of the promoter DNA-binding affinities of the I112A/F123A mutant of TipAL in the presence of thiostrepton. The corresponding result of WT TipAL (also displayed in Fig. 4*F*) is shown here for a comparison. The data were plotted using a base 10 logarithmic scale for the *x* axis and were fitted with a one-site binding model using the program GraphPad Prism7.

Fluorescence polarization assays (FPAs) were performed to test the structure-based hypothesis that α6–α7 plays a key role in communication between the ligand-binding and DNA-binding regions. TipAL, TipAN, and a truncated protein consisting of TipAN and the α6–α7 region (TipAH7, TipAL residues 1–140) were purified for the first assessment, and all of them were found to be dimers in solution (Fig. 3*A*). The affinities with the *tipA* promoter DNA (*ptipA*) were measured as 44.0 ± 5.1, 380.2 ± 56.0, and 318.5 ± 58.5 nm for TipAN, TipAL, and TipAH7, respectively (Fig. 3*B*), indicating *ptipA*-associating affinities of TipAL and TipAH7 significantly lower than the *ptipA*-associating affinity of TipAN. The double-cysteine mutant TipALCC was also applied for the analysis and showed a complete loss of DNA-binding ability (Fig. 3*B*). Parallel experiments were also performed for Mta and SkgA proteins, and consistent results were obtained. Full-length proteins and truncated proteins containing the α6–α7 region, MtaH7 (Mta residues 1–144), and SkgAH7 (SkgA residues 1–141), all exhibited a drastic loss of promoter DNA-binding affinity compared with the respective N-terminal constructs, and the double-cysteine mutants (MtaCC and SkgACC) did not show any specific binding with their respective promoter DNA (Fig. 3, *C* and *D*).

Based on the sequence homology shared by TipA-class proteins (14), including the α6–α7 region (Fig. 2*D* and Fig. S4), a possible structural model of unliganded full-length TipA proteins was established via an alignment at a structured part of the α7 helix of TipAS and Lmo0526 (residues 133–139 of TipA and residues 130–136 of Lmo0526) (Fig. 3*E*).

### Drug-binding induces a structural shift of the α7 helix for DNA binding

Because the above structural and biochemical analyses indicated that the TipAS domain inhibited TipAN–DNA binding by directly hindering access of DNA to the HTH motif in the apo state, we further investigated how drug binding induces activation. Eight residues within the α6–α7 region are reportedly involved in the TipA–thiopeptide interaction (14), half of which (Phe^123^, Tyr^131^, Val^135^, and Trp^139^) are highly conserved in the TipA family, and their corresponding residues in unliganded SkgA and Lmo0526 participate in the dimeric interaction described above. Importantly, Phe^123^, Phe^126^, Tyr^131^, Val^135^, and Trp^139^ were shown in van der Waals contact with thiopeptides, the latter three of which make key contributions to the thiopeptide recognition of TipA on the α7 helix (14) (Fig. 4, *A* and *B*). Therefore, we sought to first investigate changes in the α7 helix upon ligand binding.

The homologous α6 helices exhibit a relatively ordered and conservative structure in SkgA, Lmo0526, and ligand-bound TipAS structures, so the three structures were aligned at α6 helices for comparison (residues 111–121 of Lmo0526, residues 114–124 of SkgA, and residues 113–123 of TipAL). As a result, the α7 helix and α6–α7 linking loop of thiopeptide-bound TipAS exhibit distinctive positions and directions relative to the α6 helix compared with those of SkgA and Lmo0526 structures (Fig. 4*C*), which suggests that a structural shift of the α7 helix occurs upon drug binding to release DNA-binding domains. Further, by a close-up view of the interacting residues, Phe^123^ and Phe^126^ of ligand-bound TipAS were observed to show significant positional discrepancies with their corresponding residues in SkgA and Lmo0526, indicating that thiopeptides may induce a shift of the α7 helix via a redirection of the loop region induced by interactions with Phe^123^ and Phe^126^ (Fig. 4*D*).

Mutagenesis and biochemical analyses were further performed to test the structural hypothesis that a shift of the α7 helix would be promoted by interactions of thiopeptides with Phe^123^ and Phe^126^. F123A and F123A/F126A mutants of TipAL were produced, and their thiopeptide-binding affinities were assessed by isothermal titration calorimetry (ITC) as 7.12 ± 0.95 and 13.20 ± 1.36 μm, respectively. The affinity for WT TipAL was 5.23 ± 0.84 μm (Fig. 4*E*). F123A and F123A/F126A mutants both showed only a slight decrease in thiopeptide affinity, ∼1.4- and 2.5-fold lower than the thiopeptide affinity of the WT, which agrees with the results of a previous report indicating that the two sites do not make a key contribution to the drug recognition of TipA (14). Next, *ptipA*-associating affinities of WT TipAL and the two mutants in the presence of thiostrepton were measured by FPA as 30.2 ± 3.7, 227.6 ± 25.9, and 317.1 ± 51.4 nm, respectively (Fig. 4*F*). By thiostrepton binding, TipAL gained an ∼12.6-fold increase in *ptipA* affinity, whereas the *ptipA* binding of F123A or F123A/F126A did not show such a significant rise in the presence of thiostrepton (Figs. 3*B* and Fig. 4*F*). Collectively, these biochemical results indicated that although mutations in Phe^123^ and Phe^126^ caused no significant decrease in thiopeptide-binding affinity, they highly disrupted thiopeptide-induced activation of TipA–*ptipA* binding.

### A structural switch of the α6 helix is involved in transcriptional activation

In terms of the overall structure, such a structural shift of the α7 helix with a fixed α6 helix will lead to excessively close contact between TipAS and TipAN domains, which is still not reasonable for TipAN–*ptipA* interaction. Because the Ile^112^ residue in the loop N-terminal to the α6 helix reportedly also interacts with the thiopeptide and its corresponding residue in apo SkgA is involved in the dimeric interface (Figs. 2*D* and 4*A*), we further sought to analyze whether the α6 helix also undergoes structural changes upon drug binding. The structure of Lmo0526, which exhibits a higher sequence similarity with TipAS than SkgA on the loop region and α7 helix, was used for structural comparisons with the TipAS–thiopeptide structure.

First, the two structures were aligned at the α6 helix (residues 111–121 of Lmo0526 and residues 113−123 of TipAL) to probe potential changes of surrounding loop regions upon thiopeptide binding. The results indicated that the Phe^123^–thiopeptide interaction probably leads to extension and stabilization of the α6 helix, which is also shown in Fig. 4, and more importantly, the loop N-terminal to the α6 helix may undergo a significant deflection via the Ile^112^–thiopeptide interaction (Fig. 5*A*). Next, the structures were aligned at the ordered part of the α7 helix with relatively high identity (residues 130–136 of Lmo0526 and residues 133–139 of TipA) to evaluate variations of in the α6 helix. A significant discrepancy was observed in the orientations of α6 helices between the two structures, suggesting a drug-induced structural deflection of the α6 helix.

A two-site TipAL mutant, I112A/F123A, was then constructed to verify the structural hypothesis that the α6 helix would undergo a deflection upon drug binding. Its thiopeptide-binding affinity was measured by ITC as 7.51 ± 0.87 μm (Fig. 5*C*), without a significant difference from the thiopeptide-binding affinity of WT and F123A. However, FPA results indicated that I112A/F123A in the presence of thiostrepton showed a further lower *ptipA* affinity of 685.3 ± 97.9 nm (Fig. 5*D*), ∼23-fold smaller than the *ptipA* affinity of the WT TipAL. Taken together, the above structural and biochemical results consistently suggested that thiopeptide binding promotes a structural switch of the α6 helix for transcriptional activation.

## Discussion

Because the worldwide emergence of bacterial antibiotic resistance poses a major threat to public health, investigations on different bacterial resistance strategies are of great urgency for the development of new antimicrobial drugs (31). TipA-class transcriptional regulators compose a large family with ∼2,500 members from 1,934 species currently in the Pfam database (Fig. 1*A*). This family constitutes a sensitive autoregulated MDR system in that the MDR functional elements as drug sensor, transcriptional activators, and drug neutralizers are all integrated within a single *tipA* gene and executed by its two translation products (14). Although the molecular mechanisms of DNA binding of its MerR-like domain and antibiotic recognition of the TipAS domain have been independently well-elaborated (14, 18, 20, 24, 26), how drug binding promotes its transcriptional activation remains largely unclear (Fig. 1*A*).

The α6–α9 region involved in antibiotic recognition was revealed to be flexible loops in apo-state TipAS (14, 18), but it may result from a lack of the N-terminal part, especially a coiled-coil α5 helix that contributes to both dimerization and stabilization. Both the reported SkgA structure and the homologous Lmo0526 structure showed a structured conformation of α6–α7 helices (Fig. 1 and Figs. S2 and S3), indicating the structural difference of this region between full-length and C-terminal isoforms. Interestingly, our findings revealed that in addition to the coiled-coil region, the α6–α7 region is also involved in the dimeric interface and is stabilized via the interaction with the N-terminal DNA-binding domain in the unliganded state (Fig. 1 and Fig. S3). The interacting residues include some putative drug-binding and DNA-binding residues (Fig. S4), suggesting inhibited DNA-binding and drug-binding abilities of unliganded TipA-class regulators. The conformation of the α6–α7 region was confirmed by analysis of double-cysteine mutants of SkgA, Mta, and TipAL (Fig. 2*A*). Remarkably, α6–α7 helices exhibit an unexpected orientation that may lead to a distinctive ligand-free position of the TipAS domain compared with effector domains of other bacterial MDR regulators such as BmrR (Figs. 2*B* and 3*E*), which suggests that TipA proteins adopt a different drug-induced activation mechanism.

TipAN and MtaN are reportedly constitutively transcriptionally active in the absence of the TipAS domain (19, 26), and consistent results were indicated by our FPA assays (Fig. 3, *B* and *C*), suggesting an autoinhibition–disinhibition mechanism for transcriptional activation of the TipA-class proteins. Based on our structural and biochemical analyses, the α7 helix exhibits negative charges situated within the intermediate cleft between two MerR-type HTH domains (Fig. 2, *C* and *E*, and Fig. S5). Our FPA results showed that the α6–α7 helices largely inhibited the promoter DNA interaction of the HTH motifs in that α6–α7 containing truncations (TipAH7, MtaH7, and SkgAH7) exhibited ∼4–7-fold lower DNA-binding affinity than those of N-terminal truncations (TipAN, MtaN, and SkgAN) (Fig. 3, *B–D*). Moreover, structural prediction via the alignment of α7 helices revealed that the rest of the TipAS domain occupies the putative space for bound DNA and therefore sterically hinders the entry of promoter DNA into HTH domains in the apo state (Fig. 3*E*). The structural hypothesis is supported by the FPA results that full-length TipAL, Mta, and SkgA exhibited even lower affinities, and specific DNA interactions were completely abolished for their double-cysteine mutants whose α6–α7 region was fixed by disulfide bonds (Figs. 2*A* and 3*A*–*D*).

With such an apo-state assembly of DNA- and drug-binding domains, TipA proteins require a relatively large conformational change to release DNA-binding domains for transcriptional activation. Several residues within the α6–α7 region were reportedly employed in thiopeptide recognition of TipAS (Fig. 4, *A* and *B*), inducing restructuring, stabilization, and formation of a lid to finally sequester thiopeptides (14). In the context of full-length TipA proteins, although α6–α7 helices remain an ordered conformation without high flexibility, as shown in the structure of the TipAS protein, the surrounding loops still exhibit long and flexible conformations (Fig. 2*B*), which contribute to the conformational switch of the TipAS domain upon drug binding. By structural analysis, we found that three key residues in the surrounding loop region that are stabilized by the interaction with the DNA-binding domain (Fig. 1 and Figs. S3 and S4), Ile^112^, Phe^123^, and Phe^126^, may undergo a large structural shift upon antibiotic binding to drive an overall switch of the TipAS domain. Drug-induced variations of Phe^123^ and Phe^126^ may coordinately lead to both a sharp switch, as well as stabilization of the α7 helix (Fig. 4, *C* and *D*), and those of Ile^112^ and Phe^123^ could cooperatively result in redirection and stabilization of the α6 helix (Fig. 5, *A* and *B*). Mutations on these sites showed no significant influence on the thiopeptide-binding affinity of TipAL but resulted in a great reduction (∼7–26-fold) of its *ptipA*-associating affinity in the presence of thiostrepton (Fig. 4, *E* and *F*, and 5, *C* and *D*), which indicated their crucial roles in conformational switching for transcriptional activation rather than drug binding.

**Figure 6.**
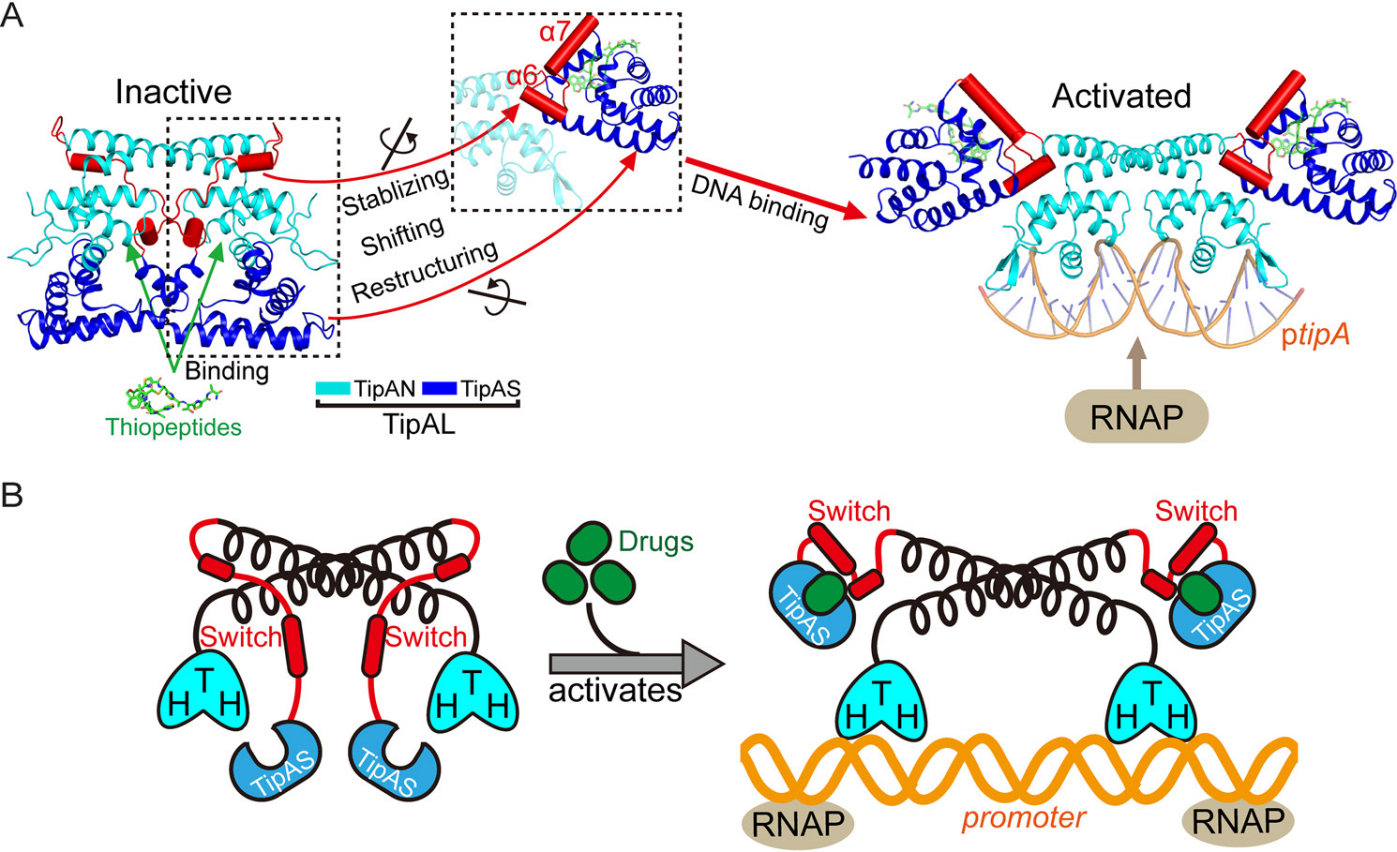
**Model representation for the structural mechanism of antibiotic-induced transcriptional activation of TipA-class proteins.** *A*, the access of promoter DNA (*ptipA*) to N-terminal HTH motifs is sterically hindered by the unliganded TipAS domains in the apo inactive state (*left panel*). Once upon antibiotic bindings, the α6–α7 switch element undergoes stabilization, restructuring, and shifting (*dotted boxes*) and thus drives a global conformational transition of TipAS domains to release the inhibition of DNA-binding. Hence, TipAN is activated to bind *ptipA* and further recruit the RNAPs to promote MDR gene transcription (*right panel*). *B*, a simplified cartoon illustration of the above established model explaining transcriptional activation mechanism of TipA-class regulators.

Based on our findings, a model was established to explain the activation mechanism of TipA-class regulators. Although promoter-DNA interaction is inhibited by the TipAS domain in the transcriptionally inactive apo state (Fig. 6, *A* and *B*, *left panels*), drug binding induces stabilization, restructuring, and a significant shift of the switch element (the α6–α7 region), which drives overall structural turnover of the TipAS domain to facilitate promoter DNA binding and subsequent recruitment of RNA polymerases (RNAPs) (Fig. 6, *A* and *B*, *right panels*).

Because TipAS confers a multiantibiotic binding capability and its residues involved in the drug-binding pocket show some sequence discrepancy among species, TipA-class regulators may respond to a different spectrum of antibiotics among evolutionarily remote bacteria. However, to date, although the autoregulated thiopeptide-resistance mechanism of TipA has been well-elucidated in *S. lividans*, the physiological functions and corresponding drugs of TipA-class proteins in other species remain mostly unreported and worth further investigation and identification. In addition, because the full-length structure model of TipA-class proteins is difficult to achieve by X-ray crystallography, CryoEM analysis on the TipA–DNA–RNAP complex is awaited for a better understanding of the activation dynamics.

In summary, our findings in this study demonstrated an unexpected autoinhibition/inhibition-release mechanism for transcriptional activation of TipA-class regulators. The α6–α7 region was revealed to serve as an activating switch that is responsible for driving a conformational transition for the release of transcriptional inhibition upon antibiotic binding. The findings provide a crucial improvement for the current model of the working mechanism of TipA-class regulators and contribute to a better understanding of the bacterial MDR system.

## Experimental procedures

### Cloning, expression, and purification

The encoding genes of full-length and truncated *S. lividans* TipA (accession no. AAB27737.1), *C. crescentus* SkgA (accession no. AAF01797.1), and *B. subtilis* Mta (accession no. CAA69863.1) were PCR-amplified and cloned into a pET-28a vector containing a His_6_ tag. All site-directed mutations were generated using the MutanBEST site-directed mutagenesis kit (Takara Bio, Dalian, China). The recombinant plasmids were sequenced and transformed into *Escherichia coli* BL21 (DE3) strains (Novagen, Darmstadt, Germany). A selenomethionine (SeMet) derivative of SkgA (SeMet-SkgA) was produced using the methionine biosynthesis inhibition method. Cells from a single transformed clone were incubated in LB medium at 37 °C until the *A*_600_ reached 0.6–0.8 and then induced using 0.5 mm isopropyl β-d-thiogalactopyranoside at 16 °C for 18 h. The cells were collected by centrifugation and lysed by sonication. The lysed cells were centrifuged at 35,000 rpm for 30 min, and the supernatants were used for purification.

Prior to biochemical use, the recombinant proteins were first loaded on a nickel–nitrilotriacetic acid column (Qiagen) with buffer consisting of 20 mm Tris-HCl, pH 7.5, 500 mm NaCl, and 5% (v/v) glycerol and eluted with 500 mm imidazole. The proteins were further purified using a HiLoad 16/60 Superdex 200 SEC column (GE Healthcare) with a buffer consisting of 20 mm Tris-HCl, pH 7.5, 50 mm NaCl, and 5% (v/v) glycerol. For crystallization, an additional purification step using a Resource-Q column (GE Healthcare) was performed. The purified proteins were concentrated using ultrafiltration to 20 mg/ml, then quick-frozen in liquid nitrogen, and stored at −80 °C.

### Crystallization, data collection, and structure determination

Crystallization was conducted using the previously described hanging-drop vapor-diffusion method (32). Diffraction quality crystals of native SkgA and SeMet-SkgA were obtained with a reservoir solution containing 13% (v/v) ethanol, 100 mm NaCl, 5% 2-Methyl-2,4-pentanediol, and 100 mm Tris-HCl, pH 7.8. The obtained crystals captured using cryoloops were soaked in cryoprotectant solution (20% (v/v) glycerol in reservoir solution) for 1 s and were immediately flash-frozen and preserved in liquid nitrogen for the diffraction experiments. Diffraction data were collected on Beamlines 17U1 and 19U1 at the Shanghai Synchrotron Radiation Facility at 100 K (33). The diffraction data sets were then processed using HKL-3000 (HKL Research Inc., Charlottesville, VA, USA).

The SeMet-SkgA structure was solved by the single-wavelength anomalous dispersion phasing technique using the AutoSol program as implemented in PHENIX (34). AutoSol found solutions, and the initial model was built automatically using AutoBuild in PHENIX and further refined using Refmac5 (35) and COOT (36). Then the model of SeMet-SkgA was utilized as the search model for molecular replacement determination of native SkgA structure using MOLREP (37). The model of SkgA was further refined at 2.50 Å resolution using Refmac5 and COOT. After several cycles of interactive refinements, we successfully established the model in the final structure. Both the coordinates and structure factors have been deposited in the PDB under accession code 7CLA. The statistical parameters for the data collection and refinement are summarized in Table 1.

### SDS-PAGE

For analysis of SkgA crystals, crystals were captured using cryoloops, washed with reservoir buffer, and then processed for separation on a 15% SDS-polyacrylamide gel followed by Coomassie Brilliant Blue staining. For analysis of double-cysteine mutants of SkgA, Mta, and TipAL (SkgACC, MtaCC, and TipALCC), WT and mutant proteins were processed with normal sample buffer and nonreduced sample buffer without redox reagent, respectively, and applied for analysis on 15% SDS-polyacrylamide gel followed by Coomassie Brilliant Blue staining.

### Size-exclusion chromatography assay

Analytical SEC assays were performed with a Superdex 200 10/300 column (GE Healthcare) on an AKTApurifier FPLC system (GE Healthcare). Purified full-length and truncated TipA proteins (40 μm) were loaded onto a column containing SEC buffer (20 mm Tris-HCl, pH 7.5, 150 mm NaCl, 1 mm DTT) for assessment. The data analysis was performed using GraphPad Prism7 (GraphPad Software, San Diego, CA, USA).

### Isothermal titration calorimetry

The ITC binding studies of the nosiheptide (Sigma) with WT TipAL and its mutants were performed using an ITC200 (GE Healthcare) at 298 K using a previously described protocol (38), with 60 μl of nosiheptide solution in the injector cell and 260 μl of protein solution in the sample cell. The injection volumes were set to 20 μl for all experiments, and two consecutive injections were separated by 2 min to reset the baseline. The control experiments, in which nosiheptide was titrated against the buffer, were also conducted, and the results were subtracted from the results of each standard experiment for adjustments. Three replicates were performed for each reaction. The final data were processed with a one-site fitting model using Microcal-Origin (OriginLab Corp, Northampton, MA, USA).

### Fluorescence polarization assay

FPAs were performed in buffer consisting of 20 mm Tris-HCl, pH 7.5, 200 mm NaCl and 5% (v/v) glycerol at 298 K using a SpectraMax M5 microplate reader system (Molecular Devices, San Jose, CA, USA). The wavelengths of fluorescence excitation and emission were 486 and 525 nm, respectively. 26-bp dsDNA from the −10 and −35 regions of the *tipA*, *mta*, and *skgA* promoters with the sequences TTGCACCTCACGTCACGTGAGGAGGC, TTGACCCTAACGTTGCGTGATTGTTT, and TTGACCCTCACGCCACGTCAGACCTG were synthesized with a 5′-carboxyfluorescein label for the respective assays with TipAL, Mta, and SkgA proteins. For assays with thiostrepton supplementation, proteins were incubated with thiostrepton (Sigma) at a 1:1 ratio for 15 min prior to mixing with DNA in wells. Each well of a 384-well plate contained 10 nm of a fluorescently labeled DNA probe and different amounts of proteins with a final volume of 80 μl. DNA-free controls were included, and three replicates were performed for each reaction for each assay. The fluorescence polarization Δ*P* (in mP units) was calculated and fitted according to a one-site specific binding model using GraphPad Prism7 (GraphPad Software) with the function Δ*P* = Δ*P*_max_[protein]/(*K_d_* + [protein]).

### Sequence and structural analyses

Structure-based multiple sequence alignments were performed using the programs Clustal Omega (RRID:SCR_001591), ESPript (39), and WebLogo3 (40). The protein sequences used for analysis were obtained via the NCBI protein database under the accession numbers AAB27737.1 (*S. lividans* TipA), NP_391541.1 (*B. subtilis* Mta), WP_010918580.1 (*C. crescentus* SkgA), NP_464054.1 (*L. monocytogenes* Lmo0526), WP_034350175.1 (TipA-class protein from *Deinococcus radiodurans*), WP_061759197.1 (TipA-class protein from *Streptococcus pneumoniae*), WP_004454874.1 (TipA-class protein from *Clostridium difficile*), and NP_390282.2 (*B. subtilis* BmrR). The structural coordinates of TipA-class proteins except SkgA used for the structural analysis were obtained from the PDB. Structural analysis was performed and structural figures were generated using the program PyMOL (DeLano Scientific LLC, South San Francisco, CA, USA). The electrostatic distribution surfaces were generated using the APBS tool (41). The analysis of the dimeric interface of SkgA and Lmo0526 was performed using the PISA service (30) and the program PyMOL.

## Data availability

Atomic coordinates and structure factors for the reported crystal structure of *C. crescentus* SkgA have been deposited in the Protein Data Bank under accession number 7CLA.

## Supplementary Material

Supporting Information
