# Supplementary material for: Antibiotic binding releases autoinhibition of the TipA multidrug-resistance transcriptional regulator
Source: J Biol Chem. 2020 Oct 22;295(51):17865–76. doi: 10.1074/jbc.RA120.016295 (PMC7762955; doi:10.1074/jbc.RA120.016295)
Supplement: Supporting Information [file supp_295_51_17865__index.html]

Antibiotic binding releases autoinhibition of the TipA multidrug-resistance transcriptional regulator — Drug-induced activation of TipA-class regulators — Supporting Information 

# Antibiotic binding releases autoinhibition of the TipA multidrug-resistance transcriptional regulator

## Supporting Information

- Supporting Information (to be published online) - With five supplemental figures and the figure legends
