# Supplementary material for: Antibiotic binding releases autoinhibition of the TipA multidrug-resistance transcriptional regulator
Source: J Biol Chem. 2020 Oct 22;295(51):17865–76. doi: 10.1074/jbc.RA120.016295 (PMC7762955; doi:10.1074/jbc.RA120.016295)
Supplement: Supporting Information [file supp_RA120.016295_163886_1_supp_616148_q1k70h.pdf]

Antibiotic binding releases autoinhibition of the TipA multidrug-resistance transcriptional regulator

Xuguang Jiang[1,2,‡,*], Linjuan Zhang[1,‡], Maikun Teng[1] and Xu Li[1,*]

From the [1]Hefei National Laboratory for Physical Sciences at Microscale, School of Life Sciences, National Synchrotron Radiation Laboratory, University of Science and Technology of China, 96 Jinzhai Road, Hefei, Anhui, China, and the [2]Department of Cell Biology and Anatomy, Graduate School of Medicine, University of Tokyo, Tokyo, Japan

[‡]These authors contributed equally to this work

*Corresponding author. E-mail: sachem@ustc.edu.cn (X. Li); xgjiang@m.u-tokyo.ac.jp (X. Jiang).

Running title: *Drug-induced activation of TipA-class regulators*

Keywords: Multidrug resistance (MDR), transcriptional regulator, TipA, autoinhibition, activation mechanism.

Supporting Information:

Figures S1-S5

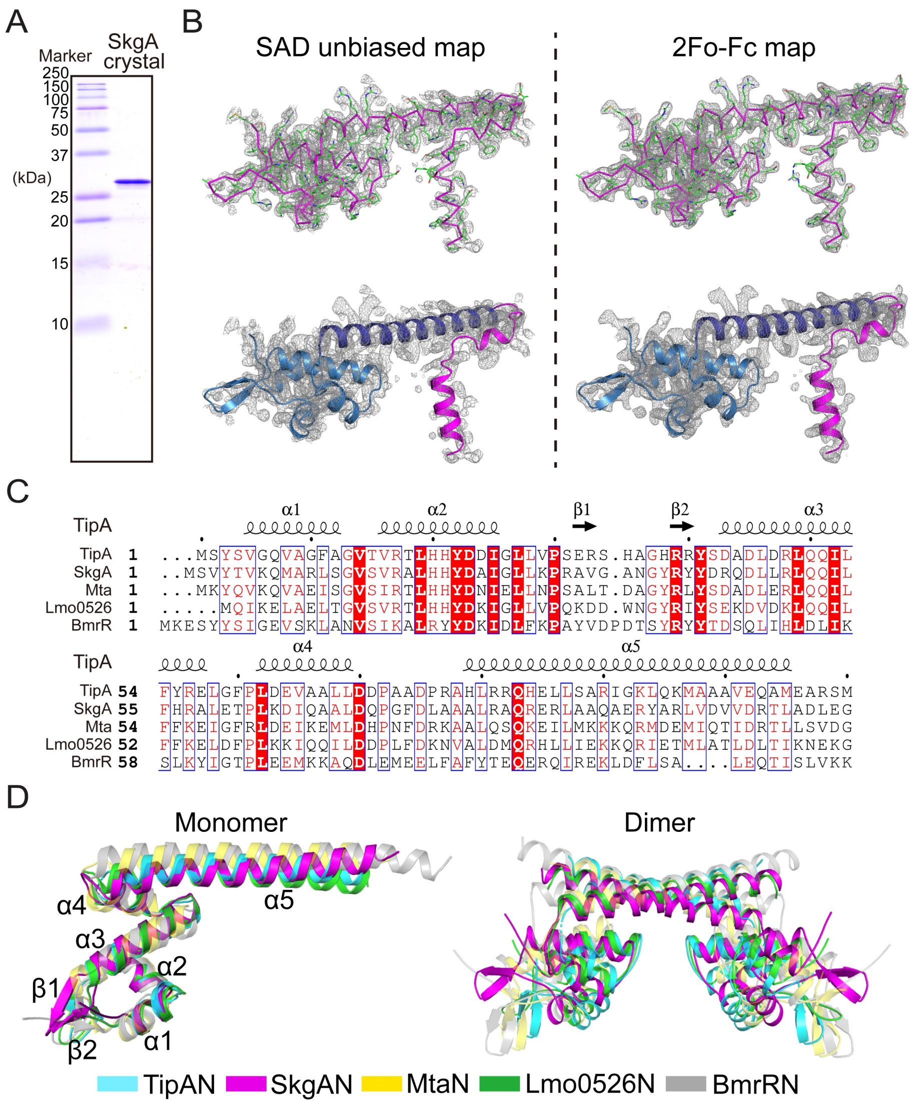

Figure S1. Crystal structure of SkgA and structural alignments of the N-terminus of four TipA-class proteins and BmrR. (A) SDS-PAGE analysis of the SkgA crystal. The crystal of SkgA was captured, washed and then assessed by SDS-PAGE. The result indicated that the proteins constituting the crystal remained as full-length SkgA without any apparent degradation. (B) Crystal structure of SkgA in the asymmetric unit. The SAD unbiased (left panel) and 2Fo-Fc density (right panel) maps are contoured at 1σ. The structure model is shown as ribbon for main chain and sticks for side chain in the upper panel and as carton in the lower panel. (C) Structure-based sequence alignment of N-terminal regions from TipA, SkgA, Mta and Lmo0526 using the programs Clustal Omega (RRID:SCR_001591) and ESPript (RRID:SCR_006587). Amino acid numbering and secondary structural elements are given according to *Streptomyces lividans* TipA. (D) Structural superposition of the N-terminus of TipA, SkgA, Mta, Lmo0526 and BmrR shown in monomer states (left panel) and dimer states (right panel). The same with TipAN and MtaN, SkgAN, Lmo0526N and BmrRN here refer to the respective N-terminal regions containing HTH and coiled-coil domains. Cα atoms of each structure were used for the superposition in the PyMOL.

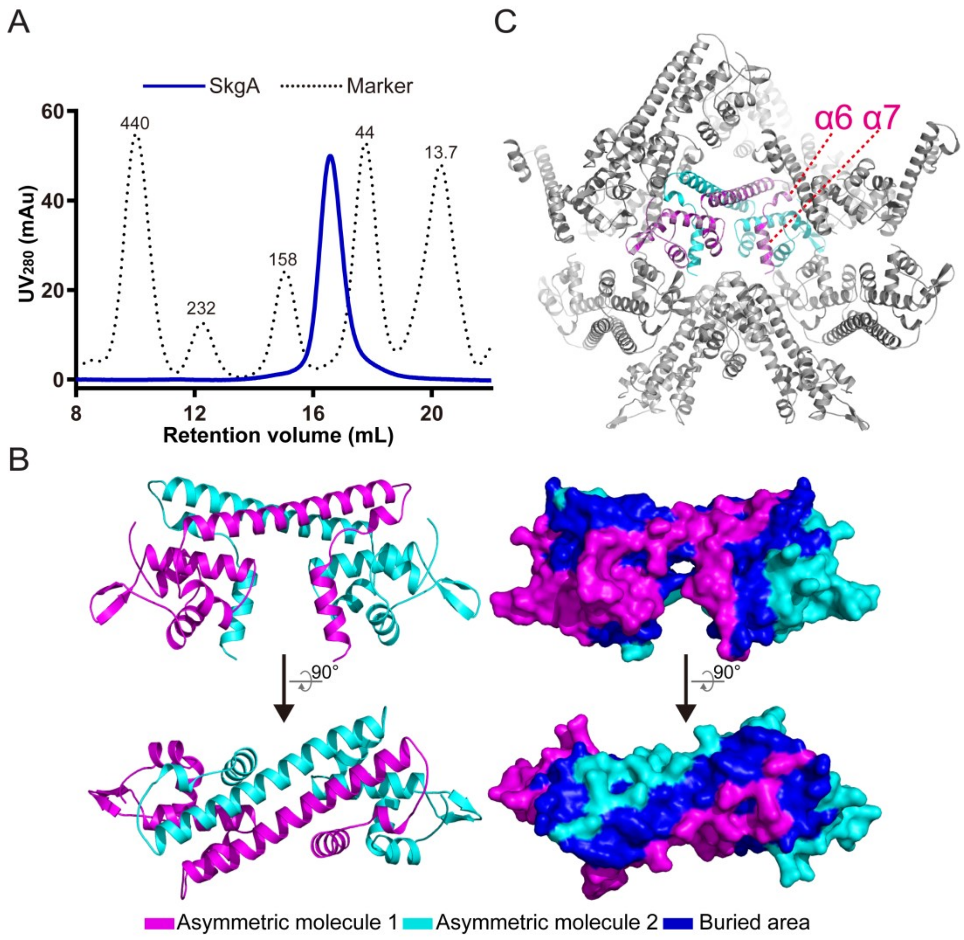

Figure S2. Analysis of the SkgA dimer. (A) Size exclusion assay analyzing full-length SkgA protein used for crystallization. Standard marker is indicated by dotted lines with molecular size (kDa) of each protein peaks marked. (B) Structure of the SkgA dimer. The dimer is constructed by two monomers from two adjacent asymmetric units. Protein surface is shown in the right panel with buried area colored in blue. (C) Symmetrical mates generated within 4Å around one asymmetric unit of the SkgA structure. No significant crystal contact toward the α6-α7 region from other symmetrical mates was observed.

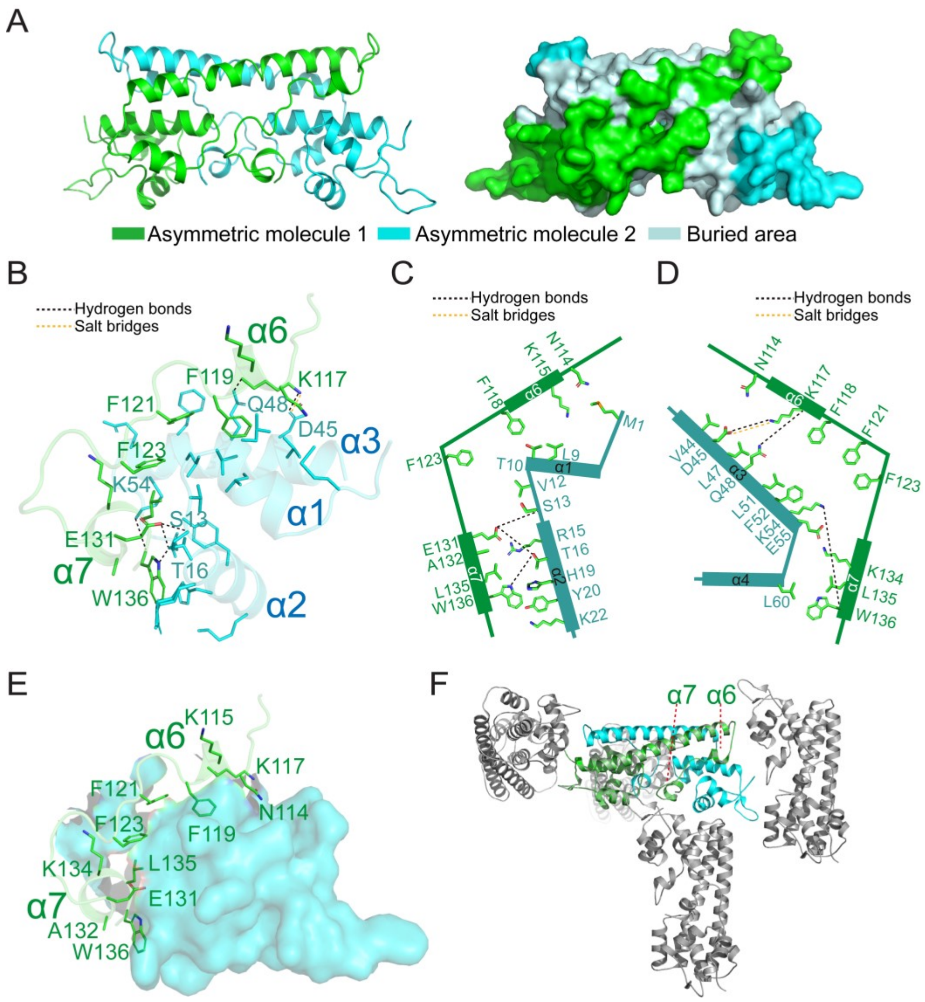

Figure S3. Basic architecture of *L. monocytogenes* TipA-class protein Lmo0526. (A) Structure of the Lmo0526 dimer. The dimer is constructed by two monomers from two adjacent asymmetric units. Protein surface is shown in the right panel with buried area colored in lightblue. (B) Interactions between the α6-α7 region and N-terminal DNA-binding domain. Hydrogen bonds and salt bridges formed between residues are indicated by black and yellow dotted lines, respectively. The other labeled residues are involved in hydrophobic interactions. (C) Schematic diagram showing the interactions between the α6-α7 region and α1-α2 region in the Lmo0526 dimer. (D) Schematic diagram showing the interactions between the α6-α7 region and α3-α4 region in the Lmo0526 dimer. (E) The α6-α7 region is involved in the dimeric interface. The α6-α7 region of one protomer interacted with the N-terminal DNA-binding domain of the other protomer. Amino acid residues of the α6-α7 region involved in the interaction are labeled with side chains shown as sticks. (F) Symmetrical mates generated within 4Å around one asymmetric unit of Lmo0526 structure. No significant crystal contact toward the α6-α7 region from other symmetrical mates was observed. Crystal structure of Lmo0526 (PDB code: 3QAO) was used for the structural analysis. The analysis of the dimeric interface was performed using the PISA service (RRID:SCR_015749) and the program PyMOL.

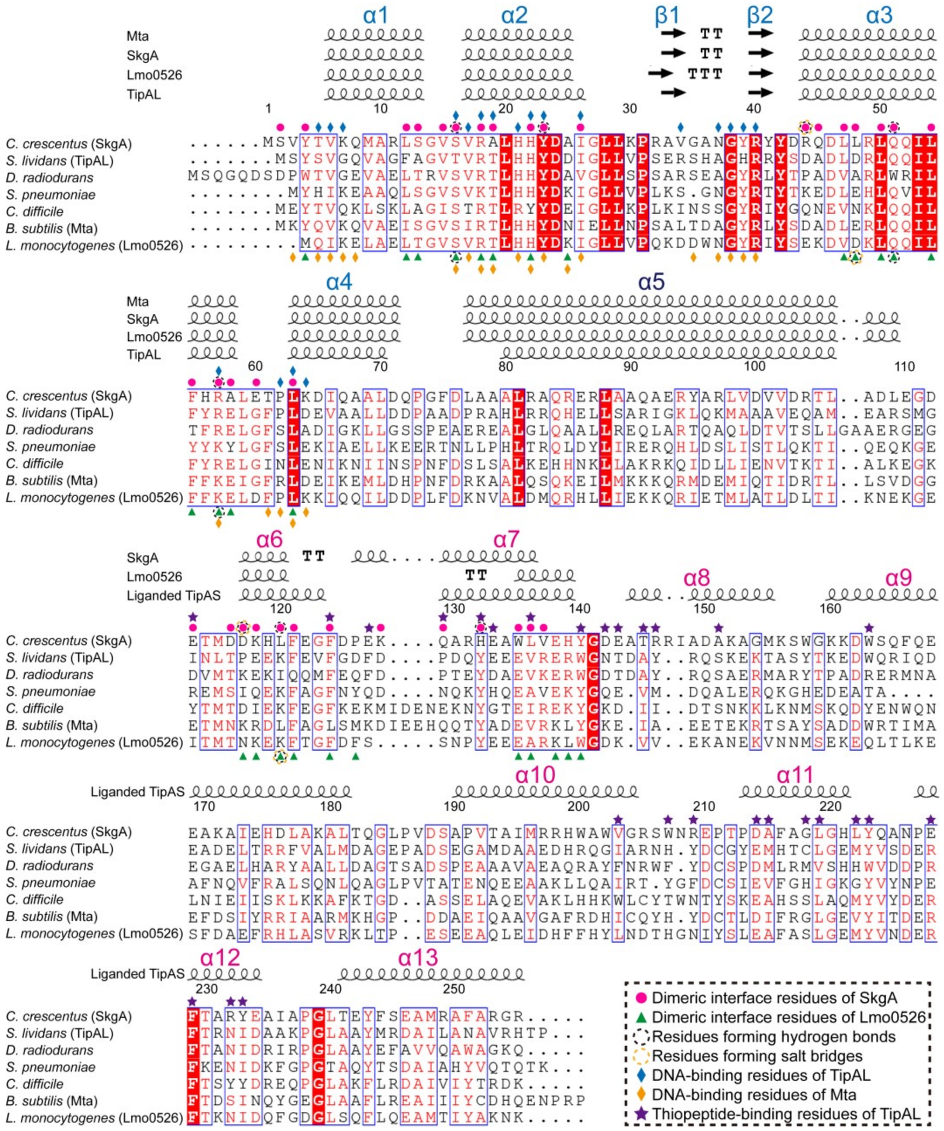

Figure S4. Structure-based multiple sequence alignment of the TipA-class regulators. Sequences of *C. crescentus* SkgA, *S. lividians* TipAL, *B. subtilis* Mta, *L. monocytogenes* Lmo0526 and their homologs from model organisms were used for the alignment. Amino acid numbering is given according to SkgA and the corresponding second structural elements of SkgA, Lmo0526 and TipA were given above the sequences. The second structure of C-terminal TipAL was shown according to the liganded TipAS structure (PDB code: 2MC0) with stabilized α6-α7 helices. Thiopeptide-binding residues of TipA are indicated by purple stars. Residues forming hydrogen bonds and salt bridges are indicated by black and yellow circled dot, respectively. DNA-binding residues of TipAL and Mta are indicated by blue and yellow rhombuses, respectively. Residues involved in the dimeric interface of SkgA and Lmo0526 are indicated by magenta circles and green arrowheads, respectively. The programs Clustal Omega and ESPript were used for the alignment.

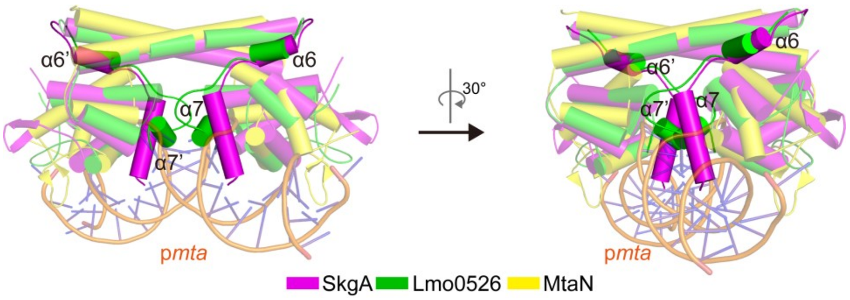

Figure S5. Putative inhibition of the Mta-DNA interaction by the α6-α7 region. Structural superposition of apo SkgA and Lmo0526 with the promoter DNA-bound MtaN (PDB code: 1R8D). Structures are represented as cartoons with cylindrical helices.
